# Optimizing Postpartum Care in Rural Communities: Insights from Women in Arizona and Implications for Policy

**DOI:** 10.1007/s10995-023-03889-w

**Published:** 2024-02-17

**Authors:** Abidemi Okechukwu, Priscilla Magrath, Halimatou Alaofe, Leslie V. Farland, Ivo Abraham, David G. Marrero, Martin Celaya, John Ehiri

**Affiliations:** 1 Mel and Enid Zuckerman College of Public Health, University of Arizona, P.O. Box 245163, Tucson, AZ 85724 USA; 2 R. Ken Colt College of Pharmacy, University of Arizona, Tucson, AZ USA; 3 University of Arizona Health Sciences (UAHS), Center for Health Disparities Research, Tucson, AZ USA; 4 Arizona Department of Health Services, Bureau of Women’s and Children’s Health, 150 North 18Th Avenue, Suite 320, Phoenix, AZ 85007 USA

**Keywords:** Postpartum, Rural, Structural determinants, Social support

## Abstract

**Objectives:**

Optimal postpartum care promotes healthcare utilization and outcomes. This qualitative study investigated the experiences and perceived needs for postpartum care among women in rural communities in Arizona, United States.

**Methods:**

We conducted in-depth interviews with thirty childbearing women and analyzed the transcripts using reflexive thematic analysis to gauge their experiences, needs, and factors affecting postpartum healthcare utilization.

**Results:**

Experiences during childbirth and multiple structural factors, including transportation, childcare services, financial constraints, and social support, played crucial roles in postpartum care utilization for childbearing people in rural communities. Access to comprehensive health information and community-level support systems were perceived as critical for optimizing postpartum care and utilization.

**Conclusions for Practice:**

This study provides valuable insights for policymakers, healthcare providers, and community stakeholders in enhancing postpartum care services for individuals in rural communities in the United States.

**Supplementary Information:**

The online version contains supplementary material available at 10.1007/s10995-023-03889-w.

## Significance

**What is already known on this subject:**

Centering postpartum policies and interventions to particular population groups necessitates a comprehensive and nuanced understanding of priority needs.

**What this study adds:**

This study sheds light on the distinct experiences and challenges influencing postpartum experiences and healthcare utilization among childbearing individuals in rural communities in the United States.

**Supplementary Information:**

The online version contains supplementary material available at 10.1007/s10995-023-03889-w.

## Introduction

The United States (US) is undergoing a maternal morbidity and mortality crisis (Hirshberg & Srinivas, 2017). In 2020, the US recorded a mortality ratio of 23.8 per 100,000 live births—its highest in the last twenty years (Hoyert, 2022); the majority of these adverse outcomes occur during postpartum (Creanga et al., 2017). The postpartum period—days and months following childbirth—offers opportunities to prevent adverse outcomes and improve long-term health for childbearing people. Acknowledging the significance of postpartum care for overall outcomes, the American College of Obstetricians and Gynecologists (ACOG) recommends that postpartum care be continuous and accessible to all (Auguste & Gulati, 2018).

Adverse postpartum experiences are intricately associated with financial constraints, discrimination, and systemic biases within healthcare, resulting in inadequate personalized and culturally sensitive care (Bellerose et al., 2022; D’Anna et al., 2018; Hall et al., 2015; Makama et al., 2021; Wouk et al., 2021) which often leads to suboptimal health outcomes (Wang et al., 2020). Similarly, rural residents are disproportionately affected by adverse outcomes compared to their urban counterparts (Merkt et al., 2021; Petersen et al., 2019). Key US policies aim to enhance access to postpartum care. The 2010 Affordable Care Act (ACA) expanded eligibility for pregnancy-related insurance, increasing postpartum care utilization in Medicaid expansion states (Myerson et al., 2020). More recently, the 2021 American Rescue Plan Act permitted states, including Arizona, to extend Medicaid coverage from 60 to 365 days after childbirth (US White House, 2021). These policies prioritize economically disadvantaged populations, potentially neglecting the unique needs of other groups, such as rural residents. To promote equity, policy interventions should be customized to effectively address the requirements of other underserved populations (Glanz & Bishop, 2010).

Literature extensively documents the postpartum needs of Black, Latino, and economically disadvantaged childbearing people (Baker et al., 2005; Bellerose et al., 2022; D’Anna et al., 2018; Sacks et al., 2022; Wang et al., 2022; Wouk et al., 2021). However, there is limited US-based research on postpartum care among rural populations. While few studies have quantitatively analyzed the effects of rural residence on postpartum care utilization and outcomes (Bryant et al., 2016; Hung et al., 2018; Interrante et al., 2022; Kozhimannil et al., 2022), narrative perspectives on lived experiences of postpartum individuals living in rural communities are underrepresented in literature (Barton & Anderson, 2021; Gjesfjeld et al., 2015.).

Since postpartum needs vary between groups, capturing and validating narratives is necessary to guide policy interventions (Jones et al., 2014; Meriwether et al., 2023). This qualitative study examined rural individuals’ postpartum experiences and needs to highlight perceived facilitators and barriers to postpartum care utilization. A nuanced understanding of these factors will identify priorities for optimized postpartum care and support for people in the rural US.

## Methods

This study formed part of an extensive qualitative Title V Supplement Maternal Health Study in Arizona, which explored maternity experiences during pregnancy, childbirth, and postpartum. We utilized a qualitative research design by conducting in-depth interviews and reflexive thematic analysis (Braun & Clarke, 2020). This study followed ethical standards in the 1964 Declaration of Helsinki and its later amendments. Ethical approval was granted by the University of Arizona Institutional Review Board (IRB Protocol Number: 1907776588).

A semi-structured interview guide with open-ended questions on pregnancy, childbirth, and postpartum was developed and pretested. Participants were recruited from September 2021 to May 2022, using a stratified, purposive sampling method, meeting specific criteria: (1) recent childbirth within three years, (2) minimum age of 18, (3) residency in an Arizona rural area, and (4) proficiency in English or Spanish, and completed consent forms and sociodemographic questionnaires on the Research Electronic Data Capture application (REDCap) (Harris et al., 2009). A diverse group of thirty individuals (n = 30) were interviewed and received $20 each, ensuring the representation of various postpartum experiences (Braun & Clarke, 2021). Researchers conducted 30–75-min in-depth interviews via Zoom, in-person, and telephone in English and Spanish for monolingual Spanish speakers. Audio recordings in Spanish were professionally translated into English and transcribed. Transcripts were analyzed using reflexive thematic analysis methodology to identify and interpret data patterns (Braun & Clarke, 2020). These iterative processes involved familiarization, initial coding using MaxQDA (VERBI Software, 2020), and reflexive thematic mapping.

## Results

The study included participants from diverse backgrounds, encompassing variations in language, culture, parity, income levels, and postpartum journeys. Table 1 demonstrates the participant demographics, all cis females, with an age range of 20 to 41 years and an average age of 30. A significant proportion of participants, approximately 80 percent, had completed a 2–4-year college degree and possessed health insurance coverage. The ensuing themes shed light on the critical priorities highlighted by the participants.

**Table 1:** Participants’ demographics

Demographics	N	Proportion (%)
Total	30	100
Mean age; years (range)	30	30 (20 – 41)
Parity		
First-time mother	5	17
Multiple children	25	83
Insurance status		
Private Insurance	10	33
Public Insurance	17	57
No Insurance	3	10
Relationship status		
Married	23	77
Civil partnership	5	17
Single	2	6
Highest level of education		
9-12th Grade	1	3
High school diploma	1	3
Some college/2 year degree	13	44
4-year college degree	11	37
Post-graduate	4	13
Race/Ethnicity		
Black/African American	4	13
Latino	8	27
Mixed race^a^	6	21
Native American/Alaskan native	1	3
Pacific Islander	1	3
White	10	33
Language		
English only	27	77
Spanish only	3	10
English and Spanish	3	10
Other*	1	3
Job status		
Paid job (full-time)	15	50
Paid job (part-time)	4	13
No job (job-seeking)	1	3
No job (not job-seeking)	5	17
Student	3	10
Other	2	7

^a^Mixed race included African American/Latino, Latino/White

^*^German

### Theme 1: Maternity Care Experiences Influence Postpartum Care Utilization

Participants reported that their experiences of childbirth and hospital discharge influenced their engagement in postpartum care services, noting that adverse childbirth experiences and impersonal treatment discouraged them from seeking postpartum care (Table 2. A). They emphasized the importance of compassionate, person-centered clinical care that respects their decisions and choices. They observed that their interactions with obstetricians tended to be brief and insufficient for fostering trust-based relationships. They also mentioned preferring care from providers of similar gender or race (Table 2. B). Additionally, some participants favored midwives over obstetricians because midwives were perceived to be more attentive to their needs and endorsed their birthing and postpartum preferences (Table 2. C), noting that impersonal provider-patient relationships prevented them from seeking information about their health and considering future healthcare engagements (Table 2. D). A common thread among individuals who reported positive experiences was the presence of health providers who provided information and engaged in discussions about childbirth and postpartum needs (Table 2. E).

**Table 2:** Summary of themes 1–4

Sections	Themes	Representative quotes
Theme 1: Maternity care experiences		
A	Experiences during childbirth and discharge inform perceptions of postpartum experiences and care utilization	“I went to the hospital when I found out I was pregnant. I had so much anxiety. I visited my doctor, and before she even saw me, it was like, “Well, you know your cervix is incompetent. So, you better schedule that cerclage soon.” However, she was doing her due diligence, probably out of deep care for me. But for me, it just felt like tearing. I was referred [to specialist care], but I chose not to go.—ID91
B	Congruence between clients and providers in race, gender, and life experiences	“My OB/GYN was a male, and just with his personality, I think he might as well have just been filling out a checklist. I don’t think he would’ve read your emotions or acted like he cared or wanted to discuss. But the two nurse practitioner midwives that worked for him; it’s just a little different feel when talking to them.”—ID37
C	Respect for preferences	“I feel like the midwife was great; she said: “This is your pregnancy. We’ll do things how you want to until there’s a medical reason why it can’t happen. And then, the obstetrician, whom I had never met until five hours before I started pushing, came in and asked, “So, what’s your plan?” And I said, “I plan on getting an epidural. I’d like to avoid getting a C-section, get the baby out safe. Keep me safe,” and he said, “All right. Let’s do it.” So, I never felt like there was any pushback for my wants.”—ID86
D	Person-centered clinical care	“It’s just that the experience is like an assembly line and mechanical. Like if you have questions or have concerns, you have to be the one that fights for that because your doctor doesn’t have enough time to be invested in you as a person.”—ID 71
E	Acknowledgment from health providers improved postpartum experiences	“I had wonderful support in every area: educator, hospital, well, the OB-GYN, and their clinic. Even the doctor congratulated me and gave me a little book with a bookmarker to read to my baby.”—ID81
Theme 2: Structural support and systems		
F	Intersections between geographical, financial, and structural factors	“We had our small amount of money and everything, but after having my baby, we had to borrow money from my sister and mother-in-law everywhere to pay for the hospital [bill]. Even now, we are getting bills for the medications that they gave me on the side. I felt frustrated and stressed. I was sad and angry. I told my husband: “We can’t get out of this. I want to work, but I want to be with my son.” Spanish—ID08
G	Transportation	“I would have gone to a different hospital if we had the option, but the closest hospital is over a hundred miles away from other hospitals, so I stayed and delivered here.—ID37
H	Wait times between appointments	“I don’t still go [to my appointments]. Those[appointments] have been easy [to attend] because they[providers] did accommodate me, and they accepted my insurance. However, we don’t have many specialists in my town, so the appointments are very far apart.”—ID4
I	Child care costs	“…you have to take double time off of work. You must pay double appointment visits. Sometimes, they want you to bring the baby versus not. So, you must pick him up from daycare and everything else and try to orchestrate all that, which means it takes double time, and you can’t do it on your lunch break.”—ID71
J	Financial barriers	“And there are some months that $60 is easy and some months that $60 isn’t.”-ID58. “…those weekly visits were expensive, even with insurance covering them. I think it was out-of-pocket close to $400 a week with those visits, and to me, that’s expensive.”—ID88
Theme 3: Health information and literacy		
K	Need for improved health literacy on pertinent postpartum issues such as postpartum depression, breastfeeding, and sleep support	“No one ever talked to or educated me on it [postpartum depression]. They just gave me the generic form that said do you have these symptoms, and I read it and checked no, no, no to the ten questions. Then nothing ever came of it.”—ID37 “I struggled a lot to breastfeed. Even after that, they [postpartum support] gave me virtual classes and constantly called me to check how I was feeling or if I needed more help. Then they called me so I wouldn’t feel lonely.”—ID3
L	Information needs to be on-demand and applicable in real-time	*“Who has time to read when you have a newborn, and you’re healing, and you’re exhausted, and trying to breastfeed, and… So yeah, not reading those”*—ID89
Theme 4: Networks of care		
M	Postpartum care should be up to a year	“I would say [postpartum care be for] a whole year. We all need so many things after having a baby: mental health, physical, lactation, and resources. There was a time when my husband and I were like, “Where do we get the insurance that is not going to eat up our whole paycheck?” So, insurance as well. I would say a whole year because everyone starts feeling slightly better. Breastfeeding, of course, you could stop at 12 months, so I feel like a whole year would be ideal for me.”—ID68
N	Providing a schedule of postpartum visits will improve postpartum care utilization	“The one thing I think I would appreciate is leaving the hospital with a regimen [or]follow-up system, saying: “You’re discharged, but here’s your next appointment with your midwife. It’s in a week or two. And then after that, we’ll see you every whatever.”—ID88
O	Provide community-based care as complements to clinical care,	“I have OB-GYN, but the doctors are just good for the [clinical] appointments, but then you go home, but it’s more comfortable having a doula come into your home so you can show what your problem is, instead of going to the doctor’s office, trying to explain, they don’t sometimes understand what you need, especially for me, it’s tough in medical words.—ID73
P	Community care providers are essential but not always affordable	“Yeah, I think I could have taken doula support. The biggest issue with doula support is that it’s usually not covered by insurance, and you have to pay out of pocket for it, which I wouldn’t have been able to do. There’s probably a lot of others that can’t afford to do that either.”—ID49

### Theme 2: Structural Support and Systems

Participants highlighted the significance of structural and health system factors in shaping postpartum care utilization. They emphasized how policy constraints and structural challenges posed significant barriers, resulting in the need to navigate complex obstacles, including geographical barriers, financial limitations, and deficient support structures (Table 2. F). Limited transportation resources emerged as a prominent obstacle, restricting the frequency of postpartum visits (Table 2. G). Despite the proximity of healthcare services, some participants acknowledged missing clinic appointments, citing that long intervals between postpartum visits made them cancel appointments as other postpartum priorities took precedence (Table 2. H). Participants perceived rural health facilities as inadequate, lacking essential infrastructure and sufficient personnel to provide specialized maternity and postpartum care when required. Access to a diverse pool of healthcare providers was essential, enhancing individuals’ perceptions of the quality of care available.

Juggling childcare responsibilities alongside parental work commitments, whether full-time or part-time, emerged as a significant issue for the participants. They stressed the importance of assistance in tending to the needs of their newborn’s older siblings, acknowledging its impact on their own healing and recuperation process. Additionally, the absence of childcare facilities posed additional challenges, especially when hospital visits did not allow for the accompaniment of older children. Participants had limited childcare options, which impeded their attendance at scheduled appointments (Table 2. I). Participants acknowledged that health insurance was a crucial safety net ensuring ongoing care and noted that they encountered frustrations if ineligible for postpartum care. The majority linked financial strain to postpartum depression, highlighting its impact on care-seeking and access to specialist services. Affordability, a subjective concept, was highlighted as a significant influence on postpartum care decisions, expressing concerns about recurring copays and service fees (Table 2. J). In addition, participants grappled with the decision to either return to work or leave employment to care for their newborn and other children. The choice between financial security and caregiving responsibilities was a recurring theme for many participants. While some individuals found resuming work beneficial for their mental well-being and readjustment, it primarily reflected financial pressures for others.

### Theme 3: Health Information and Literacy

Participants highlighted a critical need for more comprehensive information on various postpartum issues. Expressing frustration over inadequate knowledge regarding mental health, peripartum mood disorders (PMD), breastfeeding, and sleep support, participants felt unprepared to manage these concerns while alone with their infant (Table 2. K). Although most were screened for mental health during prenatal and postnatal visits and briefed on postpartum blues and depression, understanding the nuances of postpartum mental health proved challenging. Distinguishing between transient issues and those warranting medical attention was a common source of confusion. Despite experiencing some form of mental health challenge, participants felt ill-equipped to recognize PMD or seek appropriate assistance.

Participants reported receiving information from healthcare providers, primarily concerning newborn care and infant developmental milestones, alongside discussions on peripartum mood disorders. However, verbal discharge instructions often went unheeded due to physical discomfort, excitement, or lack of comprehension of medical terminologies. Reading materials provided at discharge were deemed beneficial, but time constraints hindered in-depth perusal (Table 2. L). Social media platforms and pregnancy websites emerged as alternative sources of health information for many participants. Acknowledging the empowering nature of print resources, they noted that these lacked guidance on navigating the healthcare system and accessing postpartum support. Preferences for communication channels varied, with a notable inclination toward electronic platforms providing real-time, on-demand information to address emerging concerns.

### Theme 4: Networks of Care

All participants emphasized the need for consistent postpartum care, advocating for a care duration of 6 months to 2 years (Table 2. M). They suggested that receiving a schedule of appointments upon discharge and integrating postpartum visits with infants’ appointments would enhance the continuity of postpartum care. (Table 2. N). Participants stressed the importance of community-based healthcare providers, particularly postpartum doulas and home visitors. They pointed out discrepancies between clinical and community-level support, citing challenges in service navigation and care coordination (Table 2. O). Doulas and home visitors were recognized as essential in addressing the lack of social support from family and friends. However, financial constraints often limited participants’ access to these services (Table 2. P).

### Theme 5: Social Support

Participants stressed the crucial role of social support in promoting positive postpartum experiences. Table 3 outlines their perspectives and policy considerations. They emphasized the importance of partners and community-level providers, including doulas and home visitors, in delivering personalized care. Participants also called for improved breastfeeding support and comprehensive sleep and mental health guidance. However, they highlighted financial constraints and limited access to support services as significant obstacles to comprehensive postpartum care.

**Table 3:** Theme 5: social support, representative statements, and priorities needs

Constructs of social support^1^	Definition	Categories	Presence of social support	Absence of social support	Priority needs
Emotional	Expressions of empathy, love, trust, and caring	Spouse, family and friends	“We have a small group of friends here that we meet with at least once weekly to let all our kids get together. We’re all in the same stage in life and trying to figure out how to raise kids. We all bounce ideas off each other. It works out well.”—ID25	“He [the child’s father] abandoned me, even though he initially supported me having his child. I have no support.”—ID69	⋅Training for spouses to empower them to provide the needed support ⋅Clinicians and providers need to prioritize personalized care ⋅Community-level support must consider using technology to extend emotional support to mothers at home
		Hospital-based care	“I did reach out to our lactation consultant. She accepts text messages, so I just texted her a bunch.” ID72	“Yeah. I feel like I didn’t get any medical support afterward. I think once you’re discharged from the hospital, it’s like, “Okay, you’re on your own”- ID71	
		Community-based care	“So it was nice having that support, both from moms that have nursed multiple children like, “Yes, it’s different. It’s fine. It’s normal.” A lactation consultant said, “Okay, these are your next steps. These are your options to try.”—ID86	“I called in the lactation consultant while in the hospital and was like, “This latch isn’t quite right, but I don’t know,” and she was standing like four feet from my bed, never came near us, and was like, “Well, as long as she’s getting milk, it’s fine.” She was very passive about it”.—ID86	
Instrumental	Tangible aid and service	Spouse, family and friends	“My in-laws live here in Arizona, so we had their support, and they would help watch my son if I needed a break or something. That was helpful, and my husband would help with the baby during night feedings and changing the baby. That was supportive.”—ID60	“And I didn’t want my family that lived in another state to know about my pregnancy because they’re judgmental.”—ID69	⋅Training support for intimate partners to know how best to support their partners postpartum ⋅Prioritize instrumental support for breastfeeding for everyone ⋅Prioritize policy programs such as Women Infants and Children (WIC), paid parental leave, and health insurance coverage to address inequities
		Hospital-based care	“I feel like I did have that support from even the doctor’s office, the OB-GYN, I could call them, and they would even call me to check on me to see how I was doing if I had expressed concern.”—ID81	“I should have been recommended to a midwife who cared a little more about the home situation. They also didn’t tell me how I should bathe my baby since it’s at home, which is more practical.”—SpanishID4	
		Community-based care	“…with the home health nurse, is just someone in the home to visit and check up on, make sure, like I said, breastfeeding, mental awareness, that kind of thing is all going well.”—ID29	“I gave myself everything I needed. It would’ve been helpful to have insurance pay for it [doula]because much of the support was not covered by insurance.—ID91	
		Policy/Structural	WIC was excellent; it helped me get more nutrients because fruits, vegetables, and all-wheat bread were expensive. And now, really, all we do is eat. And we love it when we can stock up on our fruits and vegetables, peanut butter, etc. That stuff helped.—ID81	“ I think I paid three grand for my doula for all the prenatal care. And then I think she did two postpartum visits afterward, and my insurance didn’t even consider touching that. I believe that many people would benefit from that if there were funding for it.”—ID25	
Informational	Advice, suggestions, and information	Spouse, family & friends	“Sometimes I’m overwhelmed. I haven’t talked to an actual psychologist, but I’ve spoken to my sister, a child psychologist. But I’ve only talked to her about things I might be feeling.”—ID10	“I wish I had talked a little bit more, maybe with someone more familiar with a baby’s sleep schedules.” ID08	⋅Individuals depend on health providers for accurate medical information ⋅Mothers need more information on sleep for mothers and babies, peripartum mood disorders, and mental health in general
		Hospital-based care	“We struggled to breastfeed originally so the pediatrician offered some support and literature. Through that information she provided, I reached out to the hospital to speak to one of the women about breastfeeding”—ID57	“I wish that there would be more options for care. Classes or seeing a midwife or nurse practitioner instead of an OBGYN in a communal setting with longer appointments where you could interact and build a relationship with your caregiver.—ID71	
		Community-based care	“With our midwife, we were on a payment plan with her for almost my pregnancy. And I recognize that even having access to someone help me, and to be able to pay that cash, is an extreme privilege that many women don’t have.—ID91	“I even juggled the idea of having a home birth, but it came down to what it cost or the requirements. Again, I didn’t have the information on it.”—ID38	

## Discussion

The study identified five key themes in rural Arizona’s postpartum experiences and care utilization. These themes highlight the influence of maternity care experiences on postpartum care utilization, the impact of structural obstacles on access, the need for comprehensive health information, the importance of sustained postpartum care, and the critical role of social support in addressing financial constraints. These findings were consistent with previous qualitative investigations of postpartum experiences in various contexts and demographics (Bellerose et al., 2022; Gjesfjeld et al., 2015; Henderson et al., 2016; Negron et al., 2013; Roman et al., 2017; Wouk et al., 2021). Our analysis underscored the idea that postpartum encounters were heavily influenced by childbirth and hospital discharge experiences. The participants’ narratives revealed negative provider behaviors, including a lack of personalized care, dismissal of prior medical experiences and preferences, and instances of disrespect that affected trust and future use of postpartum care (Bellerose et al., 2022; Wouk et al., 2021).

Utilizing postpartum care services from multiple levels of care systems, including clinics and through community-based provider visits, was crucial in enhancing the postpartum experience and overall outcomes. This study identified prominent structural barriers to postpartum care utilization, including insufficient paid parental leave, unstable health insurance coverage, the absence of financial support for crucial non-clinical services, transportation issues, limited childcare services, and links to community-level support (Barton & Anderson, 2021; Gjesfjeld et al., 2015). These findings emphasize that structural hurdles profoundly influence postpartum experiences and outcomes and shape parents’ and families’ navigation of support systems, particularly in rural communities. Qualitative and quantitative studies on postpartum care in rural areas primarily highlight the shortage of hospital-based care providers (Barton & Anderson, 2021; HRSA, 2020; Kozhimannil et al., 2020). In addition, this study illuminates the profound challenges rural childbearing people face because of the limited childcare and community-based support (Gjesfjeld et al., 2015; Henning-Smith & Kozhimannil, 2019; Kozhimannil et al., 2016; Negron et al., 2013), making the imperative for a comprehensive network of community-based providers for postpartum support.

Our findings emphasize barriers to mental health screening, diagnosis, and breastfeeding support in rural communities, underscoring the importance of structural interventions, including health insurance and community-based social support. Policy interventions targeting increased access to paid parental leave can make critical interventions more affordable for individuals and families. Insurance coverage for community and home-based services, such as doula care, could notably benefit rural postpartum individuals, enhancing access to personalized support beyond traditional clinic-based settings.

### Implications for Policy, Program, and Practice

For adequate postpartum support, prioritize comprehensive training for partners and household members of postpartum individuals, integrating technology-driven solutions at the community level for extended emotional support. Clinic-based providers play a vital role in identifying care needs, with flexibility in scheduling appointments to accommodate various demands. Integration with community-level caregivers, such as doulas and home visitors, is essential for a seamless continuum of care (Barton & Anderson, 2021; HRSA, 2020). Promoting instrumental support for breastfeeding is essential for childbearing people. Health authorities must enable insurance reimbursements for community-based providers, particularly in rural communities, where recent public health insurance expansions still need to address care needs adequately (Myerson et al., 2020). For example, expanding health insurance coverage may not improve the shortage of providers in rural communities because access barriers for economically disadvantaged individuals may differ from those encountered by rural communities. Implementing family-centric policies that enhance affordability, including paid sick leave and flexible work hours, is critical for improving postpartum care utilization and experiences, especially for rural residents (Kortsmit et al., 2021).

The qualitative approach in this study fostered safe conversations, allowing participants to share their experiences. The reflexive thematic analysis method facilitated a comprehensive interpretation of complex social phenomena and integrated researchers’ subjective interpretations with participants’ voices, creating a comprehensive and contextually rich depiction of postpartum experiences, ensuring transparency and minimizing bias. As the sampling was non-deterministic, insights from our diverse participant group may not represent the entire population of childbearing individuals in rural US communities. This calls for caution in generalizing findings. Additionally, the subjective nature of qualitative research introduces the potential for bias in data collection, interpretation, and analysis. While efforts were made to minimize bias through reflexivity, the influence of the researchers’ perspectives and preconceptions on findings cannot be entirely ruled out. Nonetheless, these findings provide valuable insights into the nuanced needs of postpartum people in rural settings.

## Conclusion

These findings underscore the influence of maternity care experiences and structural barriers on postpartum care utilization and advocate for comprehensive person-centered postpartum care that acknowledges the need for community-level support and services and policy interventions to improve accessibility and affordability of social support services.

### Supplementary Information

Below is the link to the electronic supplementary material.

Supplementary file1 (DOCX 31 KB)
