# Supplementary material for: Optimizing Postpartum Care in Rural Communities: Insights from Women in Arizona and Implications for Policy
Source: Matern Child Health J. 2024 Feb 17;28(7):1148–59. doi: 10.1007/s10995-023-03889-w (PMC11180024; doi:10.1007/s10995-023-03889-w)
Supplement: Supplementary file 1 — Supplementary file1 (DOCX 31 KB) [file 10995_2023_3889_MOESM1_ESM.docx]

INTERVIEW GUIDE

**Optimizing Postpartum Care in Rural US Communities: Insights from Women in Arizona and Implications for Policy**

|  | **SECTION** | **QUESTIONS** |
| --- | --- | --- |
| 1 | **DEMOGRAPHY** | Sample questions: How old are you? Which county do you live in? How many childbirths have you had? How old is your youngest child? What is the highest level of education you completed? Do you consider your area of residence rural or urban? |
| 2 | **POSTPARTUM CONFIRMATION** | Congratulations on the birth of your baby! All questions in this interview refer to your most recent pregnancy.  When did you give birth? |
| 3 | **PRENATAL CARE** | 1. Did you ever visit a health provider during your last pregnancy?  - If yes, was your provider a doctor, doctor’s assistant, midwife, nurse practitioner, or other? Did they identify any problems, and were you referred to another specialist?  1. How far or close to you was the clinic where you got prenatal care?  - Why did you choose that facility? |
|  | Experience/Expectations/Challenges | 1. How was your overall experience of your prenatal care?  - Did you feel respected or allowed to decide for yourself and your baby? - Did providers give you information that was culturally and language-appropriate?  1. Did you face any challenges accessing prenatal care, including specialists and referrals to other forms of care during this pregnancy?   Prompts: How hard or easy was it to find a provider? Was it hard or easy to find someone who took your insurance? |
|  | (Birth Planning) | Were there any challenges with planning your labor & delivery, including choosing your birth location and timing?  Did anyone assist you with making those plans? |
| 4 | **BIRTH** | 1. Did you give birth at the same facility as you had prenatal care?  - If not, why? - Who assisted with the delivery of your baby? |
|  | (Experiences) | 1. Did you or your baby have any difficulties or complications? Did you feel comfortable with how your providers handled that care?   You don’t have to share specifics of the complications if you don’t want to.   1. Did your providers do anything that made your birth and discharge easier? What else could have been done? 2. What education did you receive before discharge about the postpartum period regarding things to watch out for in yourself or your baby (e.g., postpartum warning signs)? Was that information helpful? |
|  | (Health promotion during pregnancy) | During your last pregnancy, did you change any behaviors to promote health?  Prompts: diet, exercise, etc. |
| 5 | **POSTPARTUM EXPERIENCE** | 1. How have you been doing/what was your experience the first year after giving birth to your baby?  - How is your child doing now? - Have you done anything special to promote your or baby’s health, including mental health? |
|  | Postpartum care services | 1. Since the birth of your child/within the first year of the delivery of your child, have you visited a healthcare provider for yourself? Why or why not?  - What kind of health provider did you see? (An O/B, nurse practitioner? Or anyone else?) - How long after birth? How many times? Why? |
|  | Postpartum Referral | 1. Were you ever referred to an additional provider (specialist, etc.)?   How easy or hard was it to attend those appointments? |
|  | Insurance | 1. Have any changes to your health insurance or the coverage of your health care needs since childbirth? 2. How long do you think post-partum maternity care should be? Please share your reasons. |
|  | Social Support | 1. What types of support (professional and personal) did you get after childbirth?  - From whom? your partner, another family, doula, friends, home visiting services from CHW, or others in the community? Did your insurance cover the professional support? Or was it free of charge or subsidized?  1. What services/support did you wish you had or would be appropriate for you? 2. What could improve your ability to secure help and support? either from professional services or friends and family? |
|  | MENTAL HEALTH | 1. Did you receive any depression or mental health screenings before, during, or after childbirth? If so:  - What were the methods of screening? (Someone asking you questions virtually or in person with a questionnaire? Or both?) Please describe your experience. Did you feel like you could be honest in your responses? Why or why not? What would have made it easier to be honest about your feelings if not?  1. Were you ever referred to a different provider related to mental health screening (Behavioral health specialist, etc.)?   If yes, how easy or hard was it to attend those appointments?  Did you feel the care was responsive to your needs/preferences? What did you like or dislike?  Did your health insurance cover them? |
| 6 | **GENERAL CONCLUSIONS** | 1. If you could change anything about your experience with maternal care, big or small, what would it be? 2. Is there anything you want to share that we haven’t asked or think we should know? |
|  |  | Thank you so much for all the information you have given us. We are looking for mothers who can give us ideas on improving reproductive health services for women in Arizona.   - Would you like to join us for a group discussion? |
